# The evolving role of physician organizations in quality related activities

**DOI:** 10.1186/2045-4015-3-18

**Published:** 2014-05-27

**Authors:** L Gregory Pawlson

**Affiliations:** 1 6726 Tomlinson Terrace, Cabin John, MD 20818, USA

## Abstract

As the fields of quality assessment and improvement become integral parts of medical practice, the roles of National Medical Associations, and other physician organizations in these endeavors have undergone major changes in scope and intensity as well. The survey based report in this journal by Levi et al. suggests some major overall trends but also notes wide variation from country to country. In this commentary, we touch on some likely reasons for the variation seen in the focus of physician organization participation in quality activities, and offer some suggestions for why expanded involvement by physician organizations may be critical to quality efforts going forward.

## Historical evolution of quality in healthcare systems

The article by Levi and his colleagues [1] related to the role of National Medical Associations in different countries in the area of quality is perhaps most notable for its finding that there is not a clear and consistent role of NMA’s across the 22 countries that participated in the survey. Also evident was the wide variation in how different the roles are that public and private sector entities play in assessing, measuring, influencing or regulating and assuring clinical quality. Using the US as an example, I will explore why the roles of NMAs may have diverged as the current chaos of quality approaches evolved (at least in the US), and end with some thoughts about what roles NMAs and specialty societies might best play going forward.

The recognition of problems related to health care quality, and how to address them, has seemed to be rather slow and uneven both within and between countries. Some of this appears to have been a result of the way in which health care itself has developed in different areas of the world, especially in regard to public versus private models of financing, delivery and payment. In addition, with a few notable exceptions, up until fairly late in the 20^th^ century, quality in health care was assumed by most to be uniformly high and assured by professionalism within medicine, nursing and other health professions. Those like Florence Nightingale in nursing, and Ernest Codman in medicine, who empirically documented results that suggested major problems in quality within healthcare, were largely ignored or even, in some instances, vilified.

In this period, professionalism did have important impacts on quality, but this was largely restricted to reforms in medical education. For example, in the US in the early 1900′s, the Flexner report and subsequent changes in medical education led to the creation of a set of standards for health professions education. This was done first at the initial professional degree level (MD, BSN)) and later this was extended to graduate and continuing education. Thus, it is not surprising that the most common and widespread activity of NMAs today is their involvement in educational standard setting.

There are some who continue to assert that professionalism (i.e. Miles, PV et al. [2] should be the sole, or at least the predominant means of assuring quality. However, empirical evidence, from seminal reports like the 1999 report of the US Institute of Medicine [3], to the widely cited study of McGlynn et al. [4] have suggested that professionalism, while very important, is insufficient to insure the highest quality of care possible for a given level of expenditures. The growth of institutional health care delivery (hospital, integrated delivery systems) in ever larger aggregations, with many different individuals and the institutions involved, has highlighted the link between quality and the way in which care is organized and delivered.

The development of more complex delivery systems have presented a major challenge to NMAs, which are, first and foremost, dues supported organizations of professionals. While defining and promoting the concepts of professionalism, they also must advocate the interests of their members or risk being seen as irrelevant. The emergence of specialty-focused medical organizations (and with medical specialty boards gaining the primary allegiance of many physicians) has further splintered the roles of the NMAs, even within the sphere of activities usually covered within professionalism. In many cases, specialty organization have begun to set at least some clinical quality standards in the form of guidelines for their members, and the many of the specialty boards have established requirements for individual certification. While the focus of Levi et al., is specifically on NMAs, it may be useful to also include specialty societies and boards in future considerations and studies, since it would seem that the objective is to ascertain the level of control or at least the degree of influence that physician organizations – of all types - have on quality. In subsequent parts of this commentary, we will use the term “organizations of medical professionals” (OMP’s) to designate this larger sphere of influence.

In only a few of the health systems analyzed by Levi (with Germany as the clearest example) does it appear the NMAs have been a primary force in the expansion of quality activities, beyond setting and monitoring educational standards. Only in a few countries have NMAs been involved in the full spectrum of measurement, feedback, benchmarking and goal setting, and increasingly, linking performance to physician or health entity payment. In the US at least, while the AMA has played important roles (along with other physician-dominated groups) in undergraduate, graduate and continuing education, some of the subspecialty societies and boards have played broader and more extensive roles in quality management than the AMA.

Without an extensive analysis of the evolution of quality activities in each country, and the roles that were played by OMPs, it is impossible to say why these groups have not been in leadership positions, and why they have not assumed more influence across broader domains of quality improvement. However, I would offer that the factors probably included some combination of the following:

1. Many OMPs appear to have assumed a more reactive than proactive role in defining and implementing quality and quality improvement, even in the area that most OMPs controlled, that of CME. This may have been in part due to the inherent conflict between being advocates for physicians, and recognizing that there are major gaps in the quality and safety of medical care. There may also have been an assumption, especially by those who have pushed for a larger role of public funding and involvement in health care, that “he who pays the piper calls the tune” – resulting in government oversight being seen as a first line of responsibility for insuring quality of care. While some groups, as for example the American Board of Medical Specialties have now expanded professional norms to include a commitment to “lifetime continuous learning” (including recertification at defined intervals), others have remained on the sidelines.

2. The limited resources available to OMPs (as contrasted with governments, or in the US, employers and private insurers) for funding development of quality improvement activities may also have played an important role. This is especially significant because, in the past, quality reviews have often involved very time and labor intensive, and therefore expensive, reviews of paper records. In addition, t implementing and disseminating quality reports can also be quite costly.

3. Another barrier has probably been the fragmentation of physician interests from National Medical Associations to an ever increasing number of medical specialty and sub-specialty organizations, and in some cases special interest groups (in the US this includes groups based on gender, race and ethnicity).

Another set of factors influencing OMP participation has been the rather haphazard and uneven emergence of both private and public sector quality regulatory and accrediting bodies. In the US, the Joint Commission (JC) began accrediting hospitals in 1951 as a successor to the hospital minimum standards program of the American College of Surgeons, which actually got its start from the work of Codman in the early 1900s. The forces that resulted in the creation the JC included both a sincere desire to extend some of the norms of professionalism (standards) to institutional healthcare, as well as a desire to avoid government interventions in regulating quality, especially at the institutional level. The AMA, along with some of the major specialty organizations (most notably internal medicine and surgery) played vital roles in the creation of the JC, and have largely controlled its board and direction since then.

The development of public sector regulation can be traced largely to 1965 when CMS (then HCFA) was given power in the original Medicare legislation of 1965 to set “conditions of participation” for hospitals and nursing homes (but in part due to AMA opposition, NOT physician practices) in the Medicare and Medicaid programs of public insurance. The legislation also gave the JC exclusive “deemed status” with CMS, so that hospitals accredited by JC were “deemed” to meet most conditions for participation in the Medicare program. Thus the creation and expansion of the JC allowed a coalition of physician organizations to essentially control hospital accreditation, at least up to the time of the implementation of Medicare, with its hospital “conditions of participation” (i.e. standards).

By contrast, the formation of the National Committee for Quality Assurance, in 1990, whose initial board and direction was controlled by executives of health plans and large employers. It is important to note that since that time the NCQA board has evolved to be a broad based group of health care leaders with only one or two of 15 or so members being from health plans. NCQA also began early in it's existence to develop and then publicly report empirical measures of health care in its HEDIS data set. Thus the formation and evolution of NCQA did not, like the JC, involve control of accrediting bodies by organized physician groups.

On the public side, it is important to note the creation of AHRQ (which in 1989 was created as the Agency for Health Care Policy and Research-AHCPR) and the expansion of CMS’s role in assurance organizations in the quality area. The growth of AHRQ, along with the establishment of a public-private partnership organization, the National Quality Forum (whose primary role is in endorsing and promulgating measures in both the public and private sectors), and the establishment of the Institute for Healthcare Improvement (IHI-a private sector organization focused on education and implementation of quality improvement efforts) have marked a rapid expansion of quality efforts in the US.

Indeed, in the past decade, there has been a proliferation of organizations, both public and private sector, that have entered the quality arena. It would seem that at this point in time, in the US and a number of other countries, this proliferation calls for a reassessment and an attempt at some coherence, including a clarification of the role and importance of involving OMPs. To ascertain further directions for OMPs in quality, it may be helpful to look at where the field of quality in healthcare is heading. For example,

1. Quality improvement is increasingly seen to be a critical component of health care delivery

2. There is a trend towards larger and more complex delivery systems in most countries,

3. The government’s role in financing and payment is large in most countries, and increasing in others (like the US)

4. The practice of medicine is becoming ever more complex and rapidly changing.

5. Even in healthcare systems that are totally government financed and delivered, patients and the healthcare professionals, along with the public at large, remain key stakeholders

A few potential directions for OMPs include:

1. **Updating the concept of professionalism.** Professionalism is still an important and for most, a desirable contract between society and those who provide certain kinds of services like medicine. However, professionalism as a concept evolved in the era before insurance, and before the recognition of the critical interests of purchasers, payers, and taxpayers and others beyond just the doctor and patient. In recent years the American Board of Internal Medicine and the American College of Physicians, along with the some International medical organizations have worked at redefining the implicit contract between physicians and both patients and other stakeholders in health care [5]. While still lacking substantial input from non-medical stakeholders including patients, it has defined a new spectrum of professionalism which includes active involvement in Qi and a commitment to “lifelong learning” (well beyond just CME).

2. **Expanding the role of OMAs in the quality arena.** Just as some advocates for patient involvement, such as Don Berwick, have called for “nothing done to me, without me” [6], it should be recognized that health professionals as represented in OMAs, should be directly involved in and influencing (but not necessarily controlling), all aspects of quality improvement. These include the development of guidelines, measures or clinical decision support, measurement and reporting, or efforts at continuous professional development. This will require major rethinking by some of those controlling the processes, and a concerted leadership and coordination of roles and efforts between NMAs, medical specialty societies, and medical specialty boards.

3. **Expanding the efforts of NMAs to actively engage their members in quality improvement leadership and activities-** as well as in trying to shape and influence the direction of outside efforts- in maintaining a strong clinical focus on QI work. For example, in the US, the AMA has recently increased its focus on quality improvement by making it one of three key strategic foci for AMA. This has resulted in major pushes by the AMA to improve care for diabetes and heart disease in close collaboration with other group, as well as in sponsorship (and convening) of the Physician Collaborative for Practice Improvement and National Quality Registry Network.

In summary, as Levi et al. [1] have noted, the roles of NMA and the expanded spectrum of physician organizations included in definition of OMPs is very diverse. Their involvement and support for quality of care related efforts is critical if we are to truly integrate quality into the everyday practice of medicine, and in health care in general.

## Competing interests

The author has no competing interests.

## Authors’ information

Dr. Pawlson MD, MPH has been Executive Director for Quality Innovation with Blue Cross Blue Shield Association, Executive Vice President of NCQA, and served in various roles at George Washington University, including Sr. Associate VP for Health Affairs and Chair of the Department of Health Care Sciences, where he is currently Clinical Professor of Medicine and Health Sciences and Adjunct Professor of Nursing.

## Commentary on

Levi, B, Borow, M and Gelkin, M. Participation of National Medical Associations in quality improvement activities- International comparison and the Israeli case- Israel Journal of Health Policy Research. 2014, 3:14
